# Postnatal Involution and Counter-Involution of the Thymus

**DOI:** 10.3389/fimmu.2020.00897

**Published:** 2020-05-12

**Authors:** Jennifer E. Cowan, Yousuke Takahama, Avinash Bhandoola, Izumi Ohigashi

**Affiliations:** ^1^Laboratory of Genome Integrity, Center for Cancer Research, National Cancer Institute, National Institutes of Health, Bethesda, MD, United States; ^2^Experimental Immunology Branch, National Cancer Institute, National Institutes of Health, Bethesda, MD, United States; ^3^Division of Experimental Immunology, Institute of Advanced Medical Sciences, University of Tokushima, Tokushima, Japan

**Keywords:** thymus, Myc, cyclin D1, growth, involution, aging

## Abstract

Thymus involution occurs in all vertebrates. It is thought to impact on immune responses in the aged, and in other clinical circumstances such as bone marrow transplantation. Determinants of thymus growth and size are beginning to be identified. Ectopic expression of factors like cyclin D1 and Myc in thymic epithelial cells (TEC)s results in considerable increase in thymus size. These models provide useful experimental tools that allow thymus function to be understood. In future, understanding TEC-specific controllers of growth will provide new approaches to thymus regeneration.

## Introduction

The thymus is the primary site of T cell development. It is a specialized environment that fosters the production of a diverse T cell repertoire, allowing T cells to recognize and eliminate foreign antigen but remain tolerant to self. The thymic epithelial cells (TEC)s educate developing T cells, where cortical (c)TECs select for those expressing functional receptors, whilst medullary (m)TECs deplete those with potential specificity to the body's own cells (1). TECs provide essential signals to T cell precursors that drive their migration, differentiation, proliferation, and survival. Correspondingly, developing thymocytes provide signals to drive TEC differentiation and organization. This process is termed thymic crosstalk (1, 2). Impaired TEC development results in severe T cell immunodeficiency (3–5).

## Thymic Involution

The thymus is the first organ to undergo aged-related involution and at an accelerated rate relative to other tissue (6). The process of thymic involution is evolutionarily conserved in all vertebrates (7). The organ undergoes rapid growth during development, peaks in size around adolescence and begins to decline with age; with the initiation of involution beginning as early as birth and no later than the onset of puberty in humans and mice (6). This thymic regression includes reductions in thymic mass, loss of thymic structure, and disorganization to thymic architecture, consequently resulting in diminished thymocyte numbers and reduced naïve T cell output (6, 8, 9). In addition to chronic age-related involution, the thymus can undergo acute atrophy under conditions of physiological stress, such as infection (10), pregnancy (11, 12), and cancer treatments (13).

Stressed-induced thymic involution results in decreased naïve T cell output and compromised host immunity, and is commonly reversible, with recovery of size and function after the insult is removed (6, 14, 15). This acute involution involves rapid reductions in proliferation and enhanced apoptosis of developing thymocytes. These impairments in developing T cells can be a direct effect of infection on thymocytes (16). However, acute thymic involution can also be a consequence of the infectious agent on TEC, which in turn drives thymocyte death (16, 17). An example of the latter is seen with synthetic dsRNA Poly(I:C) treatment, which mimics a viral infection and rapidly triggers thymic involution in mice. This acute thymic atrophy is mediated by type I IFN responses, that do not act directly on the T cell precursors (17).

Signals between TEC and thymocytes are bi-directional, with defects and alterations in the haemopoietic component of the thymus also having dramatic consequences on TEC development and maintenance. The importance of lymphoid-epithelial cell interactions for thymic architecture was established in mice lacking mature T cells, that present with severely impaired mTEC development (18). Early stage blocks in T cell development, such as in human CD3ε transgenic mice, show severely disrupted thymic architecture and defective cTEC and mTEC development (19), whilst mice with later stage defects in the TCR complex, such as genetic disruptions to the TCRα gene, Rag-1 deficiency or ZAP-70, have severely abrogated mTEC generation (19–21). Interesting, successful reconstitution in the adult thymus does not require thymic crosstalk in the fetal period. Thus, adult human CD3ε transgenic mice receiving WT fetal liver transplants had increased thymic size and restored thymic architecture and function (22). As thymocyte-derived signals are so essential in the establishment and maintenance of the thymic microenvironment, a role for both lymphoid cells and epithelial cells needs to be considered in the process of stressed-induced and chronic age-related involution.

Many agents have been identified to be involved in acute stress-induced thymic atrophy, including proinflammatory cytokines, steroids, and hormones. Inhibition of such agents can prevent this acute involution (14). The drivers of chronic age-related involution remain less clear, and it is unknown if the same cellular and molecular mechanisms underlie both chronic and acute involution (6). Sex hormones, increased after puberty, facilitate the chronic involution process, and castration of old mice can successfully albeit transiently restore both thymic size and function (23). Moreover, castration of young adult mice resulted in enhanced thymic regeneration following bone marrow transplantation (24). Sex steroid ablation can also enhance thymic function in humans. Prostate cancer patients undergoing sex steroid ablation therapy present with significant increases in numbers of naive T cells as a consequence of enhanced thymic function and T cell export (24). Although it is still poorly understood how sex steroids drive age-related thymic involution, atrophy is at least partially attributed to increased sex steroids at puberty (25).

## Immunological Consequences of Thymic Involution

Although aging results in widespread immunodeficiency, the direct consequences of age-related thymic involution on impaired immune function in the elderly is poorly understood. Old people are more susceptible to infection, and infections in the elderly often have higher severity compared to the young (8). Furthermore, the elderly mount poorer responses to vaccines (26) and show increased incidence of cancers (27). These phenomena are suggested to be, at least in part, a consequence of declining numbers and diversity of naive T cells emerging from the aged thymus, which in turn contributes to the shift toward memory phenotypes in the periphery (8, 26, 27).

Age-related thymic involution in mouse limits the numbers of recent thymic emigrants in the periphery (28). Moreover, in humans, using T-cell receptor excision circles (TREC) to measure thymic output, generation of new T cells was minimal in the elderly, with the frequency of TREC declining steadily with age (29, 30), although the precise age of when this reduction is initiated remains unclear (31). This was confirmed in a study that acquired thymic samples in addition to peripheral blood (32). Elderly individuals displayed a wide range of thymic functionality, measured by the frequency of double positive thymocytes (the higher the frequency of DP, the higher the functionality). When thymic function was compared to percentages of naïve T cells, reduced thymus function correlated well with decreased naïve T cell numbers. Moreover, this comparison identified a strong relationship between the low functioning thymi and the contraction of naïve CD8 T cells specifically. These studies also revealed a relationship between thymic function and the dynamics of peripheral naive T cells, where reduced thymus function was associated with shortened telomere length and aberrant activation and proliferation status in naïve CD8 T cells (32).

There are examples of viruses that aged mice are more susceptible to, as a consequence of age-related alteration to T cell responses. These include West Nile Virus, where old mice have reduced rates of survival compared to young. This increased susceptibility is a consequence of age-related defects in T cell immunity, involving both CD8 and CD4 T cell responses (33). Influenza has also been identified as an age-sensitive virus. It has been demonstrated that compromised protective immunity in aging against influenza can be a consequence of the age-associated decline in CD8 T cell repertoire diversity, resulting in “holes” in the repertoire that may impair the ability of T cells to control the virus (34).

Rejuvenation of the involuted thymus restores peripheral T cell function in aged mice and humans (24, 35). Many agents have been identified to reverse thymic atrophy, although their effects are only transient. Those include interleukin-7, sex steroids, growth hormones, and keratinocyte growth factor (KGF) (36). Sex steroid ablation can also improve immune responses. The level of restoration, however, following castration is limited by age, with enhancement of thymic function following the procedure lost between 9 and 18 months. Nine-month-old castrated males when challenged with Influenza A display restored numbers of virus specific cytotoxic CD8 T cells to levels comparable to young mice, and improved viral clearance in the lung (37). In contrast, 24-month-old castrated mice did not display restored numbers of virus-specific CD8 T cells to young levels, yet still had observed reductions in lung viral titers (37). Thus, age-associated defects in T cell immunity may, at least in part, be attributed to the diminished naïve T cell repertoires, and thymic regeneration by sex steroid ablation can restore this deficiency and improve viral immune responses, but in an age-limited manner. Moreover, sex steroid ablation must be having additional T-cell independent effects that improve viral clearance (37). Interestingly, a recent publication has suggested that restoration of thymic size in an aging host using sex steroid ablation or KGF does not confer protection to the host against West Niles Virus (38). Thus, modest increases to thymic size in aging hosts might not be sufficient to improve immune protection. The complete contribution of thymic involution to age-associated defects in T cell immunity remains to be determined.

Overall the immunological consequences of age-related thymic involution on the peripheral T cell pool potentially leaves the elderly with compromised protective immunity against pathogens. Thus, a better understanding of the molecular and cellular mechanisms underlining the causes of age-related thymic involution should offer benefits to the aging population in multiple clinical settings. It also needs to be considered, however, that age associated immune system defects may also be due to the aging of the hematopoietic cells, and not all a consequence of reduced thymic function. The general physiological consequences of aging to the host also needs to be considered when exploring the attributes of compromised protection against infectious agents in the elderly.

## The Role of TECs in Thymic Involution

It has been reported that the transfer of bone marrow cells or early T cell progenitors isolated from young mice does not restore thymic function in aged recipients, whereas the transplantation of fetal thymi into aged hosts exhibits thymocyte development equivalent to a young host. Thus, indicating that thymic stromal cells, rather than hematopoietic cells, drive thymic involution (15, 39–41). The reduction in size of the aged thymus is accompanied with reduced TEC numbers (9). Postnatal reductions in expression of the Forkhead box N1 (Foxn1) transcription factor is sufficient to accelerate age related thymic involution (42, 43). Moreover, targeted overexpression of Foxn1 in adult TECs can attenuate the involution process and delay the decline in naïve T cells observed in the aging host (35). Foxn1 expression is diminished in aged stroma (44), suggesting a relationship between the decline of Foxn1 expression and age-associated thymic involution.

It has been demonstrated that genetic ablation of cTECs alone has profound effects on thymic size, and severely impaired thymocyte development (45). This complements a recent publication that explored the morphological changes in TECs with age and suggested dramatic changes to cTEC structure alone reduce thymic size (46). The authors genetically labeled TECs using conditional confetti mice to facilitate the visualization of individual TECs in cortical or medullary regions. First it was established that cTECs in the thymus of young mice have unique morphology, with extensive networks of projections estimated to engulf over 100 lymphocytes within them. Second, these cTEC projections contracted in the thymus of 12-month mice, in contrast to mTEC morphology, that is unaltered with age. As the authors suggest, this dramatic alteration to cTEC morphology may contribute to thymic involution, instead of or in addition to increased cTEC death or decreased rates of cTEC proliferation with age (46). This morphological characterization of adult cTECs further offers a possible explanation as to why conventional enzymatic digestion methods used for cTEC isolation dramatically underestimate their cellularity in the postnatal thymus (47).

In addition to morphological changes with age in cTECs, their expression of Catalase, an antioxidative enzyme, is lower compared to mTECs and lymphocytes, which results in their increased susceptibility to oxidative damage (48). Indeed, the morphological alteration of cTECs is greatest in the sub-capsular cortex, which is the region of intense proliferation activity of lymphocytes, and thus of intense metabolism (46).

Transcriptional profiling of TECs in young and aged thymi has revealed dynamic gene profiles during the initial involution process in both cTECs and mTECs, aiding the understanding of the mechanisms that govern the decline of TEC numbers with age. A transcriptional hallmark of the initiation of involution in TECs was the downregulation of genes involved in cell cycle, specifically diminished E2F3 activity, suggesting possible reductions to cell cycle progression in all aging TECs (49). The same aging-associated transcriptional changes are seen in mTECs during the initial phase of thymic involution, from 2 to 10 weeks of age (50). Furthermore, gene expression profiles of cTECs display greater alterations during involution and regeneration, than those of both their mTEC counterparts and developing T cells (23). The dynamic changes in cTEC transcriptomes following castration mediated thymic regeneration revealed that genes upregulated in expression during thymic regrowth included those involved in cell morphology, cell adhesion, and cytoskeleton remodeling (46).

The underlying mechanisms of the age-related decline in TEC numbers and function that mediate thymic atrophy remain ambiguous, although many changes to TEC biology throughout life have been documented. The pivotal role of TECs in the process of thymic atrophy identify them as an attractive therapeutic target to counter thymic involution (15). Although the morphological, metabolic and transcriptional alterations with age are more prevalent in cTECs, the significance of this has yet to be determined. One major constraint of conclusively identifying a role for cTECs in age-associated thymic involution is the limitation in the numbers of cTECs able to be isolated from the adult thymus. We hope the enlarged thymus models discussed in this review will provide useful experimental tools to isolate greater numbers of adult cTECs to perform more detailed analysis of the changes that occur in this population with age. In addition, the newly refined genetic models described below will facilitate the ectopic expression of Myc specifically in cTEC or mTEC subsets (51). This could potentially determine whether such targeted manipulation is sufficient to reverse involution and force thymus growth.

## TECs Mediate Changes to Thymic Size Throughout Life

In addition to controlling thymic atrophy, TECs regulate all changes in thymic size observed throughout life. This includes the dramatic increase seen during mouse embryogenesis, when the thymus doubles in size daily, and the continued expansion, although at a reduced rate, until 4 weeks of age, when thymic cellularity peaks. TECs also maintain this maximal size until around 8 weeks of age, when involution is initiated (9). Defects in TEC development and numbers results in diminished thymic size (52). Moreover, dramatic expansion in TEC numbers in adulthood results in simultaneous expansion in the numbers of developing lymphocytes and severe thymic hyperplasia (51, 53–55). Correspondingly, and as mentioned previously, postnatal disruption of TECs triggers accelerated thymic atrophy (42, 43). Furthermore, restricting the Foxn1 dependent TEC progenitors results in reduced thymic size during the initial formation of the thymus, and this persists into the adult period (56).

The rapid expansion in thymic size and TEC number during fetal and neonatal life is supported by TECs having unique properties that distinguish them from postnatal populations. This includes higher rates of proliferation (9, 51) and ribosomal biogenesis (51). Additionally, fetal TECs are functionally distinct compared to their adult counterparts. This includes their ability to undergo successful engraftment following intra-thymic injection into an adult host, a property lost after birth (57). In addition, fetal TECs have an exclusive ability to support development of fetal waves of gamma delta T cells (58). Moreover, it has also been suggested that TEC progenitors in the fetal and postnatal thymus differ. TECs in the adult thymus have a turnover rate of ~2 weeks (9), yet their putative self-renewal capacity and precursor- product relationships remain unclear (1, 2). A common bipotent TEC progenitor has been identified at E13.5 that can give rise to both cTECs and mTECs (59, 60). During initial thymus formation, TEC progenitors can first acquire some cTEC specific markers, before differentiating into the mTEC lineage (2, 61), but mTEC lineage restricted progenitors have also been identified in the fetal thymic environment (62). Corresponding progenitor populations in the adult thymus remain to be determined (2).

## Myc Activity in TECs is Limiting for Thymic Size and Function

**Figure 1:**
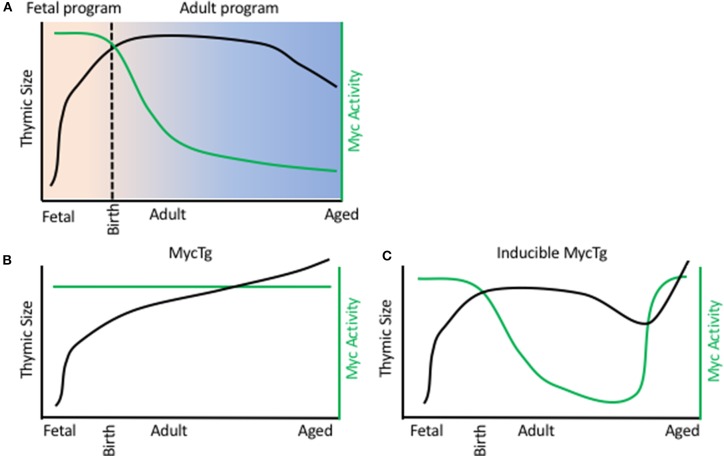
Myc activity in TEC limits thymic size. **(A)** Thymic size (black line) and Myc activity in TECs (green line) during a WT mouse life span. X axis represents age, from day of fertilization. B/C. Thymic size (black line) and Myc activity in TECs (green line) during a FoxN1 MycTg mouse **(B)** or an inducible MycTg mouse **(C)** life span.

Understanding the transcriptional controllers of fetal TECs and how they support the rapid expansion in thymic size during embryogenesis offers insight into mechanisms underlying thymic function and regeneration. Recently, transcriptional assessment of TECs through development at a single cell resolution has revealed distinct transcriptional programs of TECs at specific stages of life (51, 63–65). This included the identification of a fetal specific program comprising high levels of expression of Myc target genes, including cell cycle genes and genes involved in ribosomal biogenesis. Such genes displayed declining expression in TECs through fetal development, coordinated with reduced expression of Myc protein. Transcript levels corresponded with declining rates of cell proliferation and correlates of ribosomal biogenesis in TECs through development. It was hypothesized that this high Myc activity in fetal TECs drives the expansion in TEC numbers and consequently thymic size during early development (Figure 1A) (51). It has previously been reported that mice with conditional removal of Myc in TECs present with small thymi in adulthood, as a consequence of reduced rates of proliferation and decreased TEC numbers (66). Consistently, enhancing Myc expression in TECs resulted in a severe increase in TEC number and dramatic thymic hyperplasia in adulthood (Figure 1B). This continued Myc expression in TECs conferred expression of a fetal-specific transcriptional program, including high levels of expression of genes involved in cell cycle and ribosomal biogenesis (51). Interestingly, this ribosomal signature was unique to the Myc model, as another transgenic large thymus model, driven by cell cycle gene cyclin D1, did not present with increased transcripts of genes involved ribosomal biogenesis (51, 67).

The dramatic thymic growth conferred by the Myc transgene did not jeopardize function, similar to previous reports with cyclin D1 transgenic mice (53, 54), and such enlarged thymi also produced increased numbers of recent thymic emigrants. Furthermore, this continued Myc expression preserved the ability of TECs to engraft following intrathymic injection into adulthood. Myc overexpression did not need to be maintained throughout life to increase thymic size, with inducible expression of Myc in adult and aged TECs being sufficient to similarly promote thymic growth (Figure 1C). Although the decline in Myc activity was most striking between fetal and adult TECs, the same reductions, but at much more modest rates, could be detected transcriptionally between adult and aged cTEC populations (51). Thus, this decline in Myc activity in TECs could underlie the reduction in thymic cellularity observed during involution.

Collectively, these results identify Myc as a major regulator of a fetal specific transcriptional program of TECs. They establish a role for Myc activity in rapid thymic growth during development; provide evidence Myc expression can confer at least one functional distinct property of fetal TECs into adulthood and furthermore, confirm that Myc activity is limiting for thymic size and function (51). Upstream controllers of Myc in TECs remain to be identified and are logical candidates in modulating thymic function and regeneration.

In addition to unique transcriptional profiles of fetal TECs, the adult TECs were highly enriched for genes involved in antigen processing and presentation, along with regulation of the actin cytoskeleton and the lysosomes (51). Our results suggest an adult specific transcriptional program of TECs supports the maintenance of a functional thymus in adulthood, complementing and extending previous work (49, 63, 64).

## Cyclin D1 Overexpression in Thymic Epithelial Cells Enlarges Functional Thymus

Cyclin family molecules are key regulators for cell cycle progression that control the number of cells and the size of organs. Cyclin proteins associate and activate cyclin-dependent kinases (CDKs), serine/threonine kinases, important for the progression of cell cycle (68). Approximately 30 members of cyclin family proteins, defined by the cyclin box domain, are known in human and mice (69, 70). Among the cyclin family proteins, cyclin A, cyclin B, cyclin D, and cyclin E are canonical cyclins, which interact with cell-cycle-related CDKs and regulate cell division (71, 72). These canonical cyclins regulate distinct phases of cell cycle; cyclin A regulates S and G2 phases, cyclin B regulates M phase, cyclin D regulates G1 phase, and cyclin E regulates G1 and S phases (73–76).

Cyclin D1 is a member of cyclin D family, along with cyclin D2 and cyclin D3. Overexpression of cyclin D1 in cells results in a rapid progression from G1 to S phase, whereas inhibition of cyclin D1 prevents the entry into S phase (77). The overexpression of cyclin D1 in mouse increases the incidence of carcinoma in the mammary gland and the liver (78, 79). Correlation between elevated expression of cyclin D1 and various cancers and their poor prognosis is also noted in human (80, 81) and mouse (82).

To investigate the role of cyclin D1 in epithelial cells, Robles et al. generated transgenic mice in that cyclin D1, encoded by human *CCND1* gene, was driven by bovine keratin 5 promoter (53). Keratin 5 is primarily expressed in basal keratinocytes of the skin epidermis. It was found that these keratin 5-driven cyclin D1-transgenic (K5D1) mice exhibit epidermal hyperplasia in the skin (53). The authors also found that the thymus in K5D1 mice exhibit severe hyperplasia, which is apparent by 14 weeks of age when age-associated thymic involution is detectable in control mice, whereas the thymus in K5D1 mice at 2 weeks of age is comparable in size to that in control mice (53). In the thymus, TEC progenitors and mTECs highly express keratin 5 (54, 83), and both the cortical and medullary regions are enlarged in the thymus of K5D1 mice (53, 54).

The enlarged thymuses in K5D1 mice contain ~100-fold larger numbers of TECs compared with control C57BL/6 (B6) thymuses, and the cellularity of thymocytes in K5D1 mice reaches to ~50-fold larger numbers of that in B6 mice (67, 84). In K5D1 mice, the cellularity of cTECs is elevated similarly to that of mTECs, although keratin 5 is predominantly detectable in mTECs but not cTECs (67). It is possible that the increase in the number of cTECs is at least in part due to cyclin D1-mediated promotion of cell cycle in keratin 5-expressing TEC progenitors (54). It is additionally possible that cell cycle progression within cTEC compartment is promoted in K5D1 mice, since a remarkably elevated amount of cyclin D1 transcripts are detected in K5D1 cTECs (67). The signals provided by developing thymocytes play an important role in the development of cTECs (19, 83). Indeed, hCD3ε transgenic mice, in which early thymocyte development is primarily defective, do not exhibit thymic hyperplasia even when they are crossed with K5D1 mice to overexpress cyclin D1 in TECs (54). Thus, developing thymocytes appear to contribute to the increase in TEC cellularity in K5D1 mice.

K5D1 thymuses support thymocyte development similar to B6 thymuses, as the proportion of developing thymocytes subsets, including CD4^−^CD8^−^, CD4^+^CD8^+^, CD4^+^CD8^−^, and CD4^−^CD8^+^ thymocytes in K5D1 mice is almost comparable to that in B6 mice (67). However, mature CD4- or CD8-single positive thymocytes identified as CD69^−^Qa2^+^ are accumulated in the K5D1 thymus (84). This accumulation is possibly due to unelevated amount of sphingosine-1-phosphate (S1P), produced by unelevated number of endothelial cells in the K5D1 thymus, because S1P plays an essential role in promoting thymic egress of mature thymocytes (85). Indeed, in contrast to thymocytes, which are ~50-fold-elevated in cell number, the increase of splenic T cell number in K5D1 mice is only 2- to 3-fold of that in B6 mice (67, 84). In addition to the possible limitation in the machinery for thymic egress of mature thymocytes, limited availability of cytokines, such as Interleukin-7 (IL-7), to maintain peripheral T cells, may also limit the cellularity of the peripheral T cell pool in K5D1 mice (86).

## Thymic Epithelial Cells in Cyclin D1-Mediated Enlarged Thymus are Functionally Potent

The function of the thymus to produce self-protective T cells and to instill their self-tolerance is chiefly mediated by cTECs and mTECs, respectively. The capability of thymocyte development in K5D1 mice suggests the functional equivalence between K5D1 TECs and B6 TECs. cTECs uniquely express β5t-containing thymoproteasome important for optimal production of immunocompetent CD8^+^ T cells (87–89), whereas Aire expressed in mTECs plays a role for the establishment of self-tolerance in T cells (90). The enlarged K5D1 thymuses contain β5t^+^ cTECs in the cortex and Aire^+^ mTECs in the medulla (67). It was shown that β5t deficiency in K5D1 mice results in the impaired generation of CD8^+^ T cells in the thymus, and the loss of Aire in mTECs causes autoimmune inflammation in various tissues, including the retinas and salivary glands, in K5D1 mice (67). T cells generated in the K5D1 thymus show a proliferative response to allogenic stimulation and are unresponsive to synergic cells (67). T cells that express TCR specific for a male specific H-Y antigen are positively selected in the thymus of female K5D1 mice, whereas those T cells are negatively selected in the thymus of male K5D1 mice (54). Thus, cTECs and mTECs in the enlarged K5D1 thymus are functionally competent to produce immunocompetent yet self-tolerant T cells.

## Proteomic Profiling of Thymic Epithelial Cells Isolated From Cyclin D1 Enlarged Thymus

Isolation of cTECs and mTECs generally involves enzymatic digestion of thymic tissues. Although the thymus in one postnatal B6 mouse contains more than 1 × 10^6^ cTECs and more than 1 × 10^6^ mTECs, the number of either cTECs or mTECs isolated from one B6 mouse is typically <1 × 10^4^ cells (46, 47). However, a large number of cells (typically more than 5 × 10^5^ cells per sample) are required for current mass spectrometry-based technology of proteomic analysis, unlike transcriptomic analysis, which can be carried out from small number of cells. Using the enlarged thymus in K5D1 mice, it is possible to isolate ~2 × 10^5^ cTECs and ~2 × 10^5^ mTECs from one mouse (67). These relatively large numbers of cTECs and mTECs allow unbiased proteomic analysis.

cTECs and mTECs isolated from K5D1 mice are qualified for proteomic analysis, because RNA-sequencing-based transcriptomic profiles of cTECs and mTECs are highly similar between K5D1 mice and B6 mice (67). For example, functionally relevant genes in mTECs, including *Aire, Ccl21a*, and *Tnfrsf11a* (encoding RANK), as well as Aire-dependent and Aire-independent tissue-restricted self-antigen genes are highly detected in mTECs in an indistinguishable manner between K5D1 mTECs and B6 mTECs (67). Similarly, functionally relevant genes in cTECs, including *Dll4* (encoding DLL4), *Psmb11* (encoding β5t), *Prss16* (encoding thymus-specific serine protease or TSSP), and *Ctsl* (encoding cathepsin L), are highly detected in cTECs in an indistinguishable manner between K5D1 cTECs and B6 cTECs (67). Only a minor difference in transcriptomic profiles between K5D1 TECs and B6 TECs is the overexpression of cyclin D1 and cell-cycle-related genes (67). Another minor difference detected between K5D1 TECs and B6 TECs is the difference in the number of CD4^+^CD8^+^ thymocytes tightly associated with isolated cTECs (67). cTECs form multicellular complexes that a single cTEC completely envelops a variety number of CD4^+^CD8^+^ thymocytes (46, 91). These multicellular complexes include thymic nurse cells, which entirely encapsulate CD4^+^CD8^+^ thymocytes (91) and so cannot be fully dissociated from encapsulated CD4^+^CD8^+^ thymocytes by current technology for cTEC isolation (67). The number of enclosed DP thymocytes per one isolated cTEC is smaller in K5D1 mice than B6 mice, so that transcriptomic profiles of K5D1 cTECs is less affected than those in B6 cTECs by the signals derived from CD4^+^CD8^+^ thymocytes (67). Nevertheless, cTECs and mTECs isolated from K5D1 mice are not only functionally potent to produce immunocompetent and self-tolerant T cells but also are highly similar in transcriptomic profiles, so that cTECs and mTECs isolated from K5D1 mice are largely qualified for proteomic profiling to better understand the biology of cTECs and mTECs.

**Figure 2:**
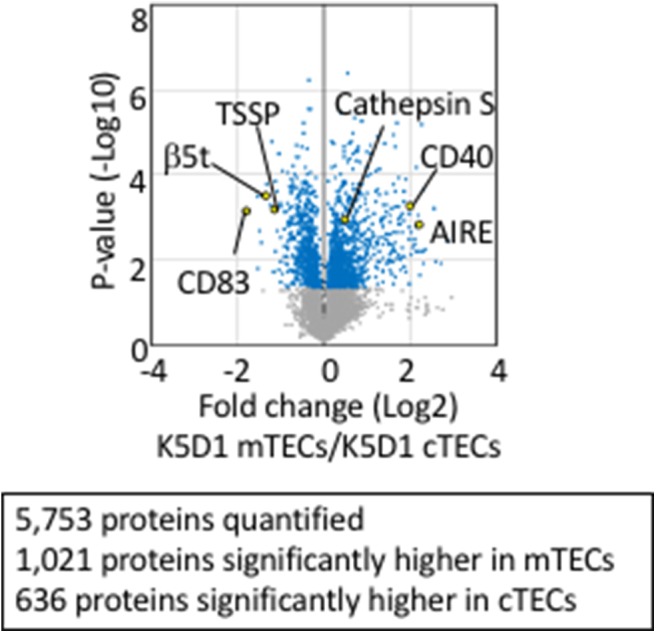
Proteomic analysis of TECs isolated from enlarged thymus. Volcano plot of TMT-based quantitative proteomes for cTECs and mTECs isolated from keratin 5 promoter-driven cyclin D1-transgenic (K5D1) mice. Detected proteins, which we have recently reported (67), are plotted as log2 fold changes (K5D1 cTECs / K5D1 mTECs) vs. –log10 *P*-values.

Tandem mass tag (TMT)-based mass spectrometry analysis is a powerful technology for unbiased proteomics that enables multiplex analysis of relative quantification of proteins (92). It successfully quantified 5,753 protein species in total in cTECs and mTECs isolated from K5D1 mice by the TMT-based quantitative proteomic analysis (67). Similar to the transcriptomic profiles, proteomic profiles show a sharp contrast between cTECs and mTECs; 636 proteins, including β5t, TSSP, and CD83, which are known to play an important role in cTECs, are significantly (*p* < 0.05) higher in cTECs than mTECs, whereas 1,021 proteins, including Aire, cathepsin S, and CD40, which are known to play an important role in mTECs, are significantly (*p* < 0.05) higher in mTECs than cTECs (Figure 2). It was noticed that secretory proteins, including cytokines and chemokines, are removed from isolated cTECs and mTECs during cell isolation procedures and are undetectable in the proteomic profiles (67).

These proteomic profiles for cTECs and mTECs have led to integrated analysis of proteomic and transcriptomic profiles in cTECs or mTECs (67). Approximately 70% of molecules detected in proteomic analysis of K5D1 TECs are detected in transcriptomic profiles of B6 TECs, whereas ~30% of molecules detected in transcriptomic profiles of B6 TECs are detected in proteomic analysis of K5D1 TECs (67). These overlapped molecules include many molecules previously reported in TECs and include molecules such as DCLK1, Avil, and Trmp5, expressed by thymic tuft cells, a recently described subpopulation of mTECs (64, 65, 67). Newly identified molecules include cathepsin D and calpain 1, abundantly expressed in cTECs, and cathepsin C, cathepsin H, and cathepsin Z, abundant in mTECs (67). These proteases may play an important role in processing self-antigens in cTECs and mTECs and in enabling TCR repertoire selection in the thymus, in addition to previously described β5t, TSSP, and cathepsin L in cTECs, and cathepsin S in mTECs. The integrated analyses of K5D1 TECs further reveal that genetic loss of β5t specifically alters the amount of individual proteasomal components in cTECs but minimally affects proteomic and transcriptomic profiles in cTECs (67).

It is interesting to note that proteomic profiles and transcriptomic profiles do not provide a proportional correlation between the amount of proteins and mRNAs in cTECs and mTECs (67). It is possible that post-transcriptional turnover of transcripts and post-translational turnover of proteins may account for the disagreement between the abundance of mRNAs and proteins (67). Thus, quantitative proteomic profiling of TECs isolated from K5D1 mice has revealed a previously unknown platform for further exploring molecular biology of TECs.

Propagation of mobilized cell lines that maintain functionally relevant molecular expression profiles has not been successful for cTECs or mTECs, so that the enlarged thymus provides a useful source of large numbers of freshly isolated cTECs and mTECs, not only for proteomic analysis but also for other analyses, including metabolomic analysis and other biochemical analyses. It is certainly important to identify MHC-associated self-peptides presented by cTECs and mTECs, which induce positive and negative selection of thymocytes to form an immunocompetent yet self-tolerant TCR repertoire.

## Why Does the Thymus Involute?

Although the immunological consequences of thymic involution have potential disadvantages to the elderly, thymic involution may have evolved for the benefit of the aging host. However, such benefits may be less favorable given current lifespans. One hypothesis is that reduced thymic activity would protect against autoimmunity (93). Seemingly at odds with this idea, accelerated thymic involution in young mice can disrupt central immune tolerance, by perturbed negative selection resulting in the release of autoreactive T cells into the periphery (94, 95). However, experimental systems could conceivably differ from naturally occurring thymic involution. Separately, it is known that some T cells in elderly mice that express markers of self-recognition are selectively retained during aging (96, 97). This could offer an explanation as to why the elderly have a predisposition to certain autoimmune conditions (94, 97), that could be mitigated by thymic involution. Overall, considerably more work is needed to determine whether the elderly are indeed at increased risk for autoimmunity, and whether reduced thymic activity may have any protective function.

It has also been suggested the involution process may conserve energy. The majority of developing thymocytes die during the stringent selection process in the thymus, where over 90% are estimated to undergo cell death (98), and the long-lived peripheral T cells established in early life can undergo homeostatic proliferation to maintain their numbers (99). Thus, energy may be best diverged into other biological processes after the T cell pool is established in early life (6).

Another possibility is that the reduced numbers of naïve T cells may also reduce the incidence of leukemias (6). Others have suggested the diminished numbers of recent thymic emigrants from the involuting thymus forces enhanced selection of the peripheral T cell repertoire and conserves the persistence of long-lived memory T cells in the periphery which are favorable to the host in old age (99, 100). If such theories are correct and involution is beneficial to the host, approaches to restore thymic size and function in old people become questionable. Therefore, a better understanding of whether thymic involution is favorable to the host will establish if thymic regeneration would be beneficial as a clinical approach.

## Conclusions and Perspectives

T cell-mediated immunity is essential throughout life. However, the thymus, where T cells are generated, involutes rapidly in early life. Postnatal thymic involution is attributable to TECs rather than thymocytes. In this review, we summarize recent findings that the decline in a fetal-specific transcriptional program of TECs controls the size of the postnatal thymus and that cTEC morphology is altered during postnatal thymic involution. These findings provide novel insights into molecular and cellular mechanisms in TECs that control thymus size during involution. Thymic involution results in reduced thymocyte development and reduced numbers of naïve T cells and so is predicted to result in immunological disadvantages, including increased incidence in infectious disease and delayed T cell reconstitution after hematopoietic stem cell transplantation (101). Therefore, prevention of thymic involution and regeneration of thymic function is likely to be useful for the maintenance and improvement of T-cell-mediated immune homeostasis. On the other hand, the increase in the number of peripheral T cells is limited in mice that carry an enlarged thymus, suggesting that merely enhancing the number of TECs and the size of the thymus is insufficient to expand the peripheral T cell pool.

In this review, we also summarize how the development of large thymus models are useful tools for understanding TEC biology. This includes the versatility of enlarged K5D1 thymi for biochemical analysis, including proteomic profiling of TECs. Using such useful tools, we can examine whether functional senescence is induced in enlarged thymi and how counter-involution in aged mice may impact health. Moreover, such models could give novel targets for manipulation to prevent or reverse thymus atrophy.

## Author Contributions

The review was written by JC and IO and was revised by YT and AB.

## Conflict of Interest

The authors declare that the research was conducted in the absence of any commercial or financial relationships that could be construed as a potential conflict of interest.
